# Excess risk attributable to traditional cardiovascular risk factors in clinical practice settings across Europe - The EURIKA Study

**DOI:** 10.1186/1471-2458-11-704

**Published:** 2011-09-18

**Authors:** Eliseo Guallar, José R Banegas, Elena Blasco-Colmenares, F Javier Jiménez, Jean Dallongeville, Julian P Halcox, Claudio Borghi, Elvira L Massó-González, Mónica Tafalla, Joep Perk, Guy De Backer, Philippe G Steg, Fernando Rodríguez-Artalejo

**Affiliations:** 1 Department of Epidemiology, Johns Hopkins Bloomberg School of Public Health, Baltimore, MD, 21205, USA; 2 Department of Medicine, Johns Hopkins School of Medicine, Baltimore, MD, 21205, USA; 3 Welch Center for Prevention, Epidemiology, and Clinical Research, Johns Hopkins Medical Institutions, Baltimore, MD, 21205, USA; 4 Area of Cardiovascular Epidemiology and Population Genetics, National Center for Cardiovascular Research (CNIC), Madrid 28029, Spain; 5 Department of Preventive Medicine and Public Health, School of Medicine, Universidad Autónoma de Madrid/IdiPAZ, Madrid 28029, Spain; 6 CIBER of Epidemiology and Public Health, Madrid 28029, Spain; 7 Medical Department, AstraZeneca Europe, Zaventem 1935, Belgium; 8 Inserm U 744, Institut Pasteur de Lille, Lille Cedex 59019, France; 9 Wales Heart Research Institute, Cardiff University, Heath Park, Cardiff CF14 4XN, UK; 10 Department of Internal Medicine, Aging and Clinical Nephrology, University of Bologna, Bologna 40100, Italy; 11 Medical Department, AstraZeneca Farmacéutica Spain SA, Madrid 28003, Spain; 12 School of Health and Caring Sciences, Linnaeus University, Kalmar 391 82, Sweden; 13 Department of Public Health, University of Gent, Gent 9000, Belgium; 14 INSERM U 698, Assistance Publique-Hôpitaux de Paris and Université Paris 7, Paris 75018, France

**Keywords:** cardiovascular disease, mortality, risk factors, control, SCORE

## Abstract

**Background:**

Physicians involved in primary prevention are key players in CVD risk control strategies, but the expected reduction in CVD risk that would be obtained if all patients attending primary care had their risk factors controlled according to current guidelines is unknown. The objective of this study was to estimate the excess risk attributable, firstly, to the presence of CVD risk factors and, secondly, to the lack of control of these risk factors in primary prevention care across Europe.

**Methods:**

Cross-sectional study using data from the European Study on Cardiovascular Risk Prevention and Management in Daily Practice (EURIKA), which involved primary care and outpatient clinics involved in primary prevention from 12 European countries between May 2009 and January 2010. We enrolled 7,434 patients over 50 years old with at least one cardiovascular risk factor but without CVD and calculated their 10-year risk of CVD death according to the SCORE equation, modified to take diabetes risk into account.

**Results:**

The average 10-year risk of CVD death in study participants (N = 7,434) was 8.2%. Hypertension, hyperlipidemia, smoking, and diabetes were responsible for 32.7 (95% confidence interval 32.0-33.4), 15.1 (14.8-15.4), 10.4 (9.9-11.0), and 16.4% (15.6-17.2) of CVD risk, respectively. The four risk factors accounted for 57.7% (57.0-58.4) of CVD risk, representing a 10-year excess risk of CVD death of 5.66% (5.47-5.85). Lack of control of hypertension, hyperlipidemia, smoking, and diabetes were responsible for 8.8 (8.3-9.3), 10.6 (10.3-10.9), 10.4 (9.9-11.0), and 3.1% (2.8-3.4) of CVD risk, respectively. Lack of control of the four risk factors accounted for 29.2% (28.5-29.8) of CVD risk, representing a 10-year excess risk of CVD death of 3.12% (2.97-3.27).

**Conclusions:**

Lack of control of CVD risk factors was responsible for almost 30% of the risk of CVD death among patients participating in the EURIKA Study.

## Background

Hypertension, dyslipidemia, smoking and diabetes mellitus are established modifiable causes of cardiovascular (CVD) disease [1,2]. However, in spite of effective interventions and widespread knowledge, the prevalence of CVD risk factors in Western populations is high and the proportion of patients with controlled risk factors is low [3,4]. It is clear that we need more effective translation strategies at the individual and the population levels to control the CVD disease epidemic.

Physicians involved in primary prevention are key players in CVD risk control strategies. Risk scoring instruments have been developed to help practitioners assess the overall CVD risk of patients and guide clinical interventions. The European Society of Cardiology has promoted the use of the Systematic Coronary Risk Evaluation (SCORE) equation to estimate the 10-year risk of CVD death, with separate equations for high and low risk regions in Europe [1,5]. It may be important for physicians to consider the reduction in CVD risk that would be obtained if all patients had their risk factors controlled according to current guidelines. As a consequence, we used data from the European Study on CVD Risk Prevention and Management in Daily Practice (EURIKA), a cross-sectional study of primary care and specialized outpatient clinics involved in primary prevention in Europe, to calculate the estimated excess risk attributable to the presence and to the lack of control of traditional CVD risk factors in usual clinical care in Europe.

## Methods

### Study population

The EURIKA Study used a cross-sectional design to estimate the degree of control of traditional CVD risk factors in clinical practice across Europe (ClinicalTrials.gov identifier, NCT00882336) [6]. EURIKA was conducted in 12 European countries (Austria, Belgium, France, Germany, Greece, Norway, Russia, Spain, Sweden, Switzerland, Turkey and the United Kingdom) from May 2009 to January 2010. Approximately 60 physicians per country were selected at random from the OneKey database, a large database containing information on the characteristics of physicians in participating countries. Physicians were selected after stratification by age, sex and specialty, among practitioners involved in CVD disease prevention in primary care centres or outpatient clinics [6].

Study participants were selected at random among those patients attending the clinics of participating physicians during the study period who were 50 years of age or older, had no clinically manifest CVD disease, and had at least one traditional CVD risk factor (hypertension, dyslipidemia, diabetes, obesity, or tobacco consumption). A total of 12,292 patients were invited to participate, of whom 7,641 (60.1%) fulfilled the inclusion criteria and consented to take part in the study. We excluded 3 patients who were younger than 50 years of age, 102 patients missing data on smoking, 13 patients missing blood pressure, 69 patients missing cholesterol levels, and 20 patients missing HbA1 c levels. The final sample size was 7,434 patients. The study protocol was approved by the appropriate clinical research ethics committees in each participating country. All patients provided written informed consent.

### Assessment of CVD risk factors

Patient information was collected from clinical records, from a standardized interview and physical exam, and from laboratory analyses of blood samples obtained during the study visit. Information on smoking was obtained from patient interviews. Blood pressure was determined at the time of the visit. A 12 h-fasting blood sample was obtained on the day of physical examination or, if not possible, on the following day. Except for blood samples from Russian centers, laboratory assays were conducted at a central study laboratory in Belgium for analysis (The Bio Analytical Research Corporation, http://www.barclab.com). Russian samples were analyzed at a local laboratory in Russia calibrated and standardized to the central study laboratory. Total cholesterol was measured by the CHOD-PAP method (Roche P-Modular) and glycosylated haemoglobin (HbA1c) was measured by ion-exchange (high-performance liquid chromatography/Menarini 8160). A 10% random sample of all study centres in each country underwent a site visit for data monitoring and quality audit.

### Definitions and treatment goals for cardiovascular risk factors

Definitions of CVD risk factors and treatment goals were based on the guidelines of the Fourth European Joint Task Force [1]. Prevalent hypertension was defined as a diagnosis of hypertension in the clinical record, current use of antihypertensive medication, or a measured blood pressure ≥ 140/90 mmHg (130/80 in patients with diabetes) at the study visit. Treatment target for hypertensive patients was a blood pressure < 140/90 mmHg (130/80 mmHg in patients with diabetes). Prevalent dyslipidemia was defined as a diagnosis of dyslipidemia in the clinical record, current use of lipid lowering medication, or a total cholesterol level ≥ 5 mmol/L (4.5 mmol/L in patients with diabetes) at the study visit. Treatment target for dyslipidemic patients was a total cholesterol < 5 mmol/L (4.5 in patients with diabetes). Prevalent diabetes was defined as a diagnosis of diabetes in the clinical record, current use of antidiabetic medication, or an HbA1 c level ≥ 6.5% at the study visit [7]. Treatment target for patients with diabetes was an HbA1 c < 6.5%.

### Statistical methods

We estimated the 10-year risk of fatal CVD disease for each patient based on the SCORE equation using data on age, sex, current smoking, total cholesterol and systolic blood pressure measured at the study visit [5]. We used the equation developed for low-risk regions for patients in Belgium, France, Greece, Spain and Switzerland, and the equation for high-risk regions for patients in Austria, Germany, Norway, Russia, Sweden, Turkey and the United Kingdom [5]. Since the SCORE equation does not incorporate diabetes as a risk factor, we modified the estimated 10-year risks for patients with diabetes assuming that the relative risks associated with diabetes were 1.89 and 2.59 for coronary events in men and women, respectively, and 2.16 and 2.83 for non-coronary CVD events in men and women, respectively [8].

To estimate risks attributable to each CVD risk factor, we recalculated the estimated 10-year risks of fatal CVD disease assuming that participants with dyslipidemia had the average total cholesterol levels of patients without dyslipidemia; that patients with hypertension had the average systolic blood pressure of patients with normal blood pressure (< 120 mmHg); that current smokers did not smoke; and that patients with diabetes did not have diabetes (that is, equal to their SCORE risk without multiplication by the relative risk for diabetes). Similarly, to estimate the risks attributable to lack of control of each CVD risk factor, we recalculated the estimated 10-year risks of fatal CVD disease assuming that participants with uncontrolled dyslipidemia were at target levels of total cholesterol; that patients with uncontrolled hypertension were at target levels of systolic blood pressure; that current smokers did not smoke; and that the risk of CVD death for diabetic patients increases by 18% for each increase in 1 percentage point in HbA1 c above target HbA1 c level [9].

Excess risks were calculated for each participant as the absolute difference between 10-year SCORE risks estimated under the observed levels of risk factors and the absence (or the control) of risk factors. Attributable risks for each participant were calculated for each participant as the ratio between the excess risk and the 10-year SCORE risk. Predicted marginal means of estimated excess and attributable risks were calculated using linear mixed models adjusted for age, sex, and country, with random intercepts for study physician. 95% confidence intervals for predicted marginal means were calculated using the delta method. Statistical analyses were performed using STATA version 11 http://www.stata.com.

## Results

The average (SD) age of study participants was 63.2 (9.0) years and the proportion of women was 51.8% (Table 1). The proportions of patients who were current smokers, hypertensive, dyslipidemic, or diabetic were 21.4%, 80.5%, 89.4%, and 29.6%, respectively. The proportions of patients with uncontrolled hypertension, uncontrolled dyslipidemia, and uncontrolled diabetes were 53.5% (66.5% of hypertensive patients), 69.8% (78.0% of dyslipidemic patients), and 19.0% (64.3% of patients with diabetes), respectively. The average 10-year risk of CVD death was 8.2%: 11.96% for men in high-risk countries, 7.08% for men in low-risk countries, 7.45% for women in high-risk countries, and 5.62% for women in low-risk countries.

**Table 1:** Characteristics of study participants, EURIKA Study 2009 - 2010

			Low risk	countries					High risk	countries			
		
	Overall	Belgium	France	Greece	Spain	Switzerland	Austria	Germany	Norway	Russia	Sweden	Turkey	UK
**N**	7,434 (100.0)	630 (8.1)	555 (7.5)	619 (8.3)	631 (8.5)	632 (8.5)	604 (8.1)	641 (8.6)	594 (8.0)	593 (8.0)	614 (8.3)	655 (8.8)	666 (9.0)
**Age (years)**	63.2 (9.0)	64.6 (8.9)	64.2 (8.8)	62.9 (8.9)	63.1 (9.8)	65.3 (9.9)	61.8 (8.6)	65.5 (8.8)	62.9 (8.5)	58.3 (7.3)	64.9 (8.6)	59.4 (7.6)	65.0 (8.9)
**Sex**													
**Men**	3,584 (48.2)	309 (49.1)	301 (54.2)	284 (45.9)	323 (51.2)	336 (53.2)	288 (47.7)	313 (48.8)	292 (49.2)	184 (31.0)	307 (50.0)	308 (47.0)	339 (50.9)
**Women**	3,850 (51.8)	321 (50.9)	254 (45.8)	335 (54.1)	308 (48.8)	296 (46.8)	316 (52.3)	328 (51.2)	302 (50.8)	409 (69.0)	307 (50.0)	347 (53.0)	327 (49.1)
**Smoking**													
**Never**	3,832 (51.6)	379 (60.2)	316 (56.9)	300 (48.5)	370 (58.6)	315 (49.8)	298 (49.3)	332 (51.8)	213 (35.9)	351 (59.2)	300 (48.9)	348 (53.1)	310 (46.6)
**Current**	1,588 (21.4)	102 (16.2)	90 (16.2)	209 (33.8)	107 (17.0)	137 (21.7)	144 (23.8)	107 (16.7)	173 (29.1)	150 (25.3)	103 (16.8)	156 (23.8)	110 (16.5)
**Former**	2,014 (27.1)	149 (23.7)	149 (26.9)	110 (17.8)	154 (24.4)	180 (28.5)	162 (26.8)	202 (31.5)	208 (35.0)	92 (15.5)	211 (34.4)	151 (23.1)	246 (36.9)
SBP (mmHg)*	135.0 (16.6)	132.6 (14.4)	133.2 (13.2)	129.8 (14.4)	133.9 (16.8)	136.1 (16.0)	135.3 (17.6)	135.3 (17.2)	136.6 (16.1)	136.5 (17.5)	140.1 (17.2)	134.4 (19.3)	136.3 (15.9)
DBP (mmHg)*	80.9 (9.9)	78.7 (8.2)	77.8 (8.9)	79.9 (8.8)	78.8 (10.2)	81.6 (10.0)	82.8 (9.7)	80.5 (9.5)	82.2 (9.9)	84.2 (10.3)	82.3 (9.7)	82.4 (11.6)	79.1 (9.9)
**Hypertension**	5,984 (80.5)	496 (78.7)	443 (79.8)	451 (72.9)	473 (75.0)	511 (80.9)	473 (78.3)	569 (88.8)	478 (80.5)	488 (82.3)	553 (90.1)	500 (76.3)	549 (82.4)
**Uncontrolled**	3,975 (53.5)	307 (48.7)	262 (47.5)	262 (42.4)	298 (47.3)	350 (55.4)	324 (53.7)	382 (59.6)	351 (59.1)	332 (56.1)	392 (64.0)	366 (56.0)	349 (52.5)
**hypertension**													
Tot. chol. (mmol/l)*	5.4 (1.1)	5.2 (1.0)	5.5 (1.1)	5.5 (1.1)	5.5 (1.1)	5.4 (1.1)	5.6 (1.2)	5.6 (1.1)	5.5 (1.1)	5.9 (1.2)	5.5 (1.2)	5.3 (1.1)	5.1 (1.2)
**Dyslipidemia**	6,646 (89.4)	560 (88.9)	508 (91.5)	582 (94.0)	581 (92.1)	568 (89.9)	544 (90.1)	577 (90.0)	548 (92.3)	532 (89.7)	550 (89.6)	509 (77.7)	587 (88.1)
**Uncontrolled**	5,185 (69.8)	376 (59.7)	401 (72.3)	438 (70.8)	459 (72.7)	433 (68.5)	457 (75.7)	486 (75.8)	436 (76.4)	484 (81.6)	424 (69.1)	446 (68.1)	345 (51.8)
**dyslipidemia**													
HbA1 c (%)*	6.1 (1.0)	5.9 (0.9)	5.9 (0.9)	6.0 (1.0)	6.0 (1.1)	6.0 (0.9)	6.0 (0.9)	6.1 (0.9)	5.9 (0.9)	6.3 (1.0)	6.1 (1.0)	6.5 (1.6)	6.0 (0.9)
**Diabetes**	2,200 (29.6)	182 (28.9)	144 (26.0)	189 (30.5)	190 (30.1)	205 (32.4)	156 (25.8)	255 (39.8)	145 (24.4)	143 (24.1)	171 (27.9)	246 (37.6)	174 (26.1)
**Uncontrolled**	1,415 (19.0)	98 (15.6)	89 (16.0)	110 (17.8)	117 (18.5)	121 (19.2)	94 (15.6)	140 (21.8)	86 (14.5)	117 (19.7)	131 (21.3)	191 (29.2)	121 (18.2)
**diabetes**													
10-y CVD risk (%)*	8.2 (9.8)	6.0 (7.2)	5.8 (6.3)	5.7 (6.7)	6.0 (6.7)	8.2 (10.1)	9.1 (10.9)	12.8 (13.2)	9.5 (9.8)	6.2 (7.7)	11.7 (12.4)	7.4 (9.3)	9.9 (10.6)
**< 5%**	3,690 (49.6)	370 (58.7)	331 (59.6)	383 (61.9)	384 (60.9)	326 (51.6)	282 (46.7)	194 (30.3)	238 (40.1)	369 (62.2)	204 (33.2)	360 (55.0)	249 (37.4)
**5-10%**	1,824 (24.5)	158 (25.1)	136 (24.5)	131 (21.2)	132 (20.9)	147 (23.3)	150 (24.8)	172 (26.8)	164 (27.6)	118 (19.9)	158 (25.7)	157 (24.0)	201 (30.2)
**≥ 10%**	1,920 (25.8)	102 (16.2)	88 (15.9)	105 (17.0)	115 (18.2)	159 (25.2)	172 (28.5)	275 (42.9)	192 (32.3)	106 (17.9)	252 (41.0)	138 (21.1)	216 (32.4)

Values in table are number (%) or mean (SD) where noted by *.

**Figure 1:**
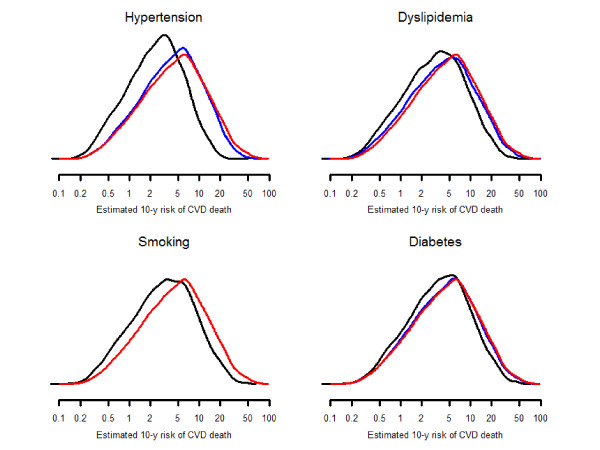
**Estimated 10-year risk of cardiovascular death calculated at current levels of blood pressure, total cholesterol, smoking, and diabetes (red line), assuming risk factors at control level (blue), and assuming absence of risk factors (black)**.

**Figure 2:**
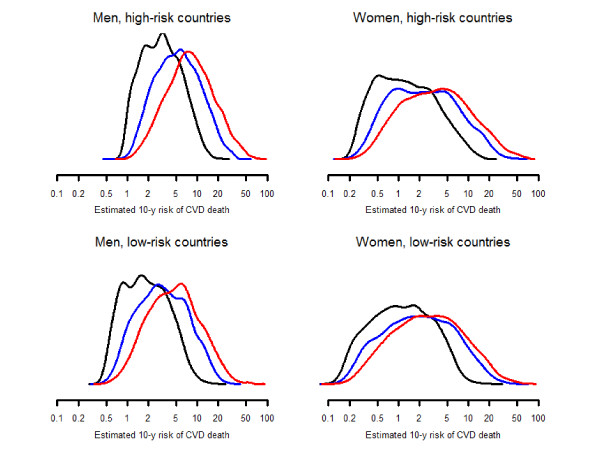
**Estimated 10-year risk of cardiovascular death calculated at current levels of risk factors (red line), assuming risk factors at control level (blue), and assuming absence of risk factors (black)**.

Hypertension, dyslipidemia, smoking, and diabetes were responsible for 32.7 (32.0-33.4), 15.1 (14.8-15.4), 10.4 (9.9-11.0), and 16.4% (15.6-17.2) of CVD risk, respectively (Table 2). These risk factors accounted for 57.7% (57.0-58.4) of CVD risk, with relatively little between country variability (between-country range 52.6 to 61.6%). The 10-year absolute excess risks of CVD death attributable to hypertension, hyperlipidemia, smoking, and diabetes were 3.49 (95% confidence interval 3.34-3.63), 1.14 (1.08-1.19), 0.92 (0.85-0.99), and 2.25% (2.12-2.39), respectively (Figure 1 and Additional File 1: Table S1). The absolute excess risk attributable to the four risk factors combined was 5.66% (5.47-5.85) (Figure 2), with a between-country range of 3.06 to 4.65% among low-risk countries and 5.39 to 8.12% among high-risk countries.

**Table 2:** Cardiovascular risk attributable to traditional cardiovascular risk factors (%), EURIKA Study 2009 - 2010

			Low risk	countries					High risk	countries			
		
	Overall	Belgium	France	Greece	Spain	Switzerland	Austria	Germany	Norway	Russia	Sweden	Turkey	UK
**Hypertension**	32.7	29.9	30.1	26.1	30.9	33.1	32.4	33.5	34.3	37.1	38.8	32.7	33.5
	(32.0-33.4)	(27.5-32.3)	(27.5-32.7)	(23.6-28.6)	(28.4-33.3)	(30.8-35.5)	(29.9-34.9)	(31.1-35.9)	(31.7-36.8)	(34.8-39.3)	(36.3-41.3)	(30.3-35.1)	(31.1-35.9)
**Dyslipidemia**	15.1	11.2	15.3	14.4	14.7	14.2	17.6	17.3	16.8	19.2	16.0	13.3	11.9
	(14.8-15.4)	(10.0-12.3)	(14.1-16.5)	(13.2-15.9)	(13.5-15.9)	(13.0-15.3)	(16.5-18.8)	(16.1-18.4)	(15.6-18.1)	(18.1-20.3)	(14.8-17.2)	(12.2-14.5)	(10.8-13.0)
**Smoking**	10.4	8.5	8.4	16.4	8.3	11.6	11.0	9.4	14.1	10.3	9.1	9.7	8.8
	(9.9-11.0)	(6.7-10.4)	(6.4-10.4)	(14.5-18.3)	(6.5-10.2)	(9.7-13.4)	(9.1-12.8)	(7.6-11.2)	(12.2-16.0)	(8.5-12.1)	(7.2-11.0)	(7.9-11.5)	(7.0-10.6)
**Diabetes**	16.4	15.7	13.9	17.1	16.7	17.5	14.7	21.5	13.6	14.5	15.1	21.8	13.7
	(15.6-17.2)	(12.9-18.5)	(10.8-16.9)	(14.2-20.1)	(13.8-19.5)	(14.7-20.3)	(11.8-17.7)	(18.7-24.3)	(10.6-16.6)	(11.9-17.2)	(12.1-18.1)	(19.0-24.6)	(10.8-16.5)
**All risk factors**	57.7	52.6	53.9	56.5	55.2	58.7	57.9	61.0	60.6	61.6	61.1	59.4	54.1
	(57.0-58.4)	(50.3-54.9)	(51.4-56.4)	(54.1-58.9)	(52.9-57.6)	(56.4-61.0)	(55.5-60.3)	(58.6-63.3)	(58.1-63.0)	(59.4-63.8)	(58.7-63.6)	(57.0-61.7)	(51.8-56.4)

Values in Table are % (95% confidence interval).

Lack of control of hypertension, dyslipidemia, smoking, and diabetes were responsible for 8.8 (8.3-9.3), 10.6 (10.3-10.9), 10.4 (9.9-11.0), and 3.1% (2.8-3.4) of CVD risk, respectively (Table 3). Lack of control of the combination of these risk factors accounted for 29.2% (28.5-29.8) of CVD risk, with a between-country range of 22.1 to 34.0%. The 10-year absolute excess risks of CVD death attributable to lack of control of hypertension, hyperlipidemia, smoking, and diabetes were 1.42 (1.31-1.53), 0.92 (0.87-0.96), 0.92 (0.85-0.99), and 0.41% (0.37-0.46), respectively (Figure 1 and Additional File 1: Table S2'). The excess risk attributable to lack of control of 4 risk factors was 3.12% (2.97-3.27) (Figure 2), with a between-country range of 1.33 to 2.50% among low-risk countries and 2.87 to 4.26% among high-risk countries.

**Table 3:** Cardiovascular risk attributable to lack of control of traditional cardiovascular risk factors (%), EURIKA Study 2009 - 2010

			Low risk	countries					High risk	countries			
		
	Overall	Belgium	France	Greece	Spain	Switzerland	Austria	Germany	Norway	Russia	Sweden	Turkey	UK
**Hypertension**	8.8	6.0	5.4	5.0	8.8	9.0	9.6	9.9	9.5	11.2	11.6	10.2	8.7
	(8.3-9.3)	(4.4-7.7)	(3.6-7.2)	(3.3-6.8)	(7.0-10.5)	(7.3-10.6)	(7.9-11.4)	(8.2-11.5)	(7.7-11.3)	(9.6-12.8)	(9.8-13.4)	(8.5-11.9)	(7.0-10.4)
**Dyslipidemia**	10.6	7.2	10.6	10.0	10.1	9.9	12.5	12.7	11.6	14.3	11.2	9.5	7.8
	(10.3-10.9)	(6.2-8.1)	(9.5-11.6)	(8.9-11.0)	(9.1-11.1)	(8.9-10.9)	(11.5-13.6)	(11.7-13.7)	(10.6-12.7)	(13.3-15.3)	(10.2-12.3)	(8.5-10.5)	(6.9-8.8)
**Smoking**	10.4	8.5	8.4	16.4	8.3	11.6	11.0	9.4	14.1	10.3	9.1	9.7	8.8
	(9.9-11.0)	(6.7-10.4)	(6.4-10.4)	(14.5-18.3)	(6.5-10.2)	(9.7-13.4)	(9.1-12.8)	(7.6-11.2)	(12.2-16.0)	(8.5-12.1)	(7.2-11.0)	(7.9-11.5)	(7.0-10.6)
**Diabetes**	3.1	2.2	2.3	2.6	3.4	2.5	2.5	2.5	2.4	3.3	3.6	6.6	2.7
	(2.8-3.4)	(1.2-3.2)	(1.3-3.4)	(1.5-3.6)	(2.4-4.4)	(1.5-3.5)	(1.5-3.5)	(1.5-3.5)	(1.3-3.4)	(2.4-4.2)	(2.5-4.6)	(5.6-7.6)	(1.7-3.7)
**All risk factors**	29.2	22.1	24.1	30.0	27.2	29.4	31.2	30.4	33.0	34.0	31.3	31.8	25.5
	(28.5-29.8)	(19.9-24.3)	(21.7-26.5)	(27.7-32.3)	(25.0-29.5)	(27.2-31.6)	(28.9-33.5)	(28.2-32.7)	(30.7-35.4)	(31.8-36.2)	(29.0-33.6)	(29.6-34.0)	(23.3-27.7)

Values in Table are % (95% confidence interval).

Absolute excess risks were particularly high among patients with current estimated risk ≥ 10% (excess risks attributable to the presence and to the lack of control of risk factors of 14.50 and 9.41%, respectively), among patients with diabetes (10.52 and 5.73%, respectively), among patients ≥ 65 years of age (9.76 and 5.12%, respectively), and among current smokers (9.09 and 6.69%, respectively) (Additional File 1: Table S3).

## Discussion

Hypertension, dyslipidemia, smoking and diabetes explained 57.7% of estimated risk of CVD death of patients 50 years of age and older with at least one CVD risk factor who attended primary and specialty clinics involved in primary prevention across Europe in the EURIKA study. Eliminating these risk factors would translate in an absolute reduction of 5.66% in the 10-year risk of CVD death. Even though EURIKA patients were under medical care, lack of control of traditional risk factors was common, and explained almost 30% of estimated risk. Control of hypertension, dyslipidemia, smoking and diabetes would reduce the estimated 10-year risk of CVD death by 3.12%. Absolute risk reductions through elimination or control of hypertension, dyslipidemia, smoking and diabetes would be particularly large in patients with high overall risk, in patients with diabetes, in elderly patients, and in current smokers.

The prevalence of CVD risk factors in the EURIKA population was high, partly as a consequence of including patients with at least one traditional CVD risk factor (hypertension, dyslipidemia, smoking, diabetes or obesity) [6]. Among subjects 50 years of age or older in Western societies, however, the prevalence of these risk factors is very high [10-13], making the EURIKA findings applicable to a wide segment of the population attending general medical care. In addition, a large fraction of EURIKA patients did not reach target levels of control [1]. These findings are consistent with earlier surveys showing highly inadequate risk factor control in patients with and without established CVD disease [3,14-18]. For instance, the EUROASPIRE III Survey, conducted in 2006 - 2007 among patients without a history of atherosclerotic disease who were treated with antihypertensive, lipid-lowering, or antidiabetic drugs in general practice in 12 European countries, found that 73.7% of patients using antihypertensive medications had blood pressure above 140/90 mmHg (above 130/80 mmHg among patients with diabetes), 69.4% of patients using lipid-lowering medications had total cholesterol above 5.0 mmol/L (above 4.5 mmol/L among patients with diabetes), and 60.1% of self-reported patients with diabetes had HbA1 c above 6.1% [3]. Our analysis of the EURIKA Study extends these findings to show that lack of risk factor control is responsible for a substantial excess risk among patients already under clinical care, highlighting the need for more effective translation of evidence-based guidelines into routine care.

Hypertension has been identified as the leading risk factor for mortality and the third cause of disability-adjusted life-years worldwide [19,20]. In the EURIKA population, elevated blood pressure was responsible for 32.7% of CVD risk. Indeed, our analyses likely underestimate the contribution of elevated blood pressure to CVD risk as we did not consider the excess risk contributed by patients with prehypertension [20]. Even though the EURIKA population was under medical surveillance and 68.5% of study patients were receiving antihypertensive medications, over 50% of EURIKA patients had measured levels of blood pressure above 140/90 mmHg. Control of blood pressure levels to target levels would reduce the estimated risk by 8.8%, with particularly high gains in Sweden, Russia, and Turkey, although there may be a degree of resistant hypertension that even after aggressive management by the clinician might not result in blood pressure being brought back to target. Beyond the well-known challenges for control of hypertension in clinical settings [21], lowering blood pressure to normotensive levels in the population will require intensive public health action to control the obesity epidemic and to promote healthy eating and exercise habits in the general population [19,20]. Our analysis of the EURIKA data emphasizes the need to combine clinic-based and population strategies to curb high blood pressure-related risk.

Dyslipidemia was responsible for 15.1% of CVD death risk in the EURIKA population, and achieving control levels of 5 mmol/L in all EURIKA participants would reduce the estimated CVD death risk by 10.6%. The impact of dyslipidemia was particularly high in Russia, a country with very high average cholesterol levels and with low rates of control. As for hypertension, control rates for dyslipidemia are low even with the availability of highly effective and safe interventions to reduce cholesterol levels [22]. Furthermore, since the benefits of cholesterol lowering therapies are independent of pre-treatment cholesterol levels or other patient characteristics [22,23], cholesterol lowering therapies could be used to achieve specific reductions in cholesterol levels (instead of targeting pre-specified levels of control) as a core component of high-risk patient management. The high proportion of patients with uncontrolled dyslipidemia in the EURIKA population and the associated excess risk further call for added clinical and public health efforts to control cholesterol levels.

With a proportion of current smokers of 21.4% in the EURIKA population, smoking accounted for 10.4% of CVD risk, with particularly high attributable risks in Greece and Norway. Furthermore, since smoking habits were identified by questionnaire without confirmation by objective biomarkers, it is likely that the proportion of current smokers and the associated attributable risk have been underestimated [24]. The benefits of quitting smoking are well documented and all current smokers should be encouraged to quit [25,26]. Primary practices and specialized clinics are important checkpoints in this process [26], although population strategies are needed to favor a smoke-free environment and an appropriate atmosphere for sustained quitting.

Diabetes was responsible for 16.4% of CVD risk in the EURIKA population, but control of diabetes would only reduce CVD risk by 3.1%. Excess risks associated with diabetes were particularly high in Turkey and Germany. While diabetes is a strong independent risk factor for CVD, the benefits of intensive glycemic control on macrovascular disease outcomes and mortality are controversial. Indeed, recent trials have shown either no significant reduction in CVD outcomes or increased mortality with intensive glycemic control [27]. In our analysis, we assumed based on observational data that the risk of CVD death for diabetic patients increases by 18% for each 1 percentage point increase in HbA1 c above target HbA1 c level [9], but this assumption will need to be modified as additional evidence accumulates on the impact of intense glycemic control on CVD outcomes and we develop more reliable estimates of the benefit of glucose control on macrovascular CVD outcomes. While attaining glycemic control targets is still a key objective of management of patients with diabetes, our data also show that substantial risk benefits can be realized in patients with diabetes by control of other cardiovascular risk factors. Ultimately, however, the main approach to diabetes risk control should be based on primary prevention of diabetes through control of the obesity epidemic and adoption of healthy lifestyles.

Our estimates of attributable risks in the EURIKA population rely on a series of assumptions. First, SCORE equations were developed to have better calibration for European populations, but there is concern that the SCORE equation may overestimate risk in many Western European countries with decreasing secular trends in CVD mortality as well as in elderly patients [28]. Calibration of the SCORE equation to country-specific CVD mortality rates has been advocated [29], but joint data on CVD risk factors and mortality rates in country-wide representative population samples were not available in most EURIKA countries. Conversely, the SCORE equation may underestimate risk in Russia and other Eastern European countries that are experiencing extremely high rates of CVD mortality [30,31]. Furthermore, the SCORE equation may underestimate risk in patients with certain risk factors not included in the equation, such as those with a sedentary lifestyle, central obesity, a family history of premature CVD, or with the presence of subclinical atherosclerosis [1]. Country specific calibration or recalibration of equations to current CVD death rates will change the estimates, although we notice that the proportion of CVD risk attributable to each risk factor and overall is relatively constant across countries, suggesting that the main conclusions of our analyses are likely to hold under revised risk equations.

Second, the EURIKA population was already under clinical care and a high proportion of EURIKA patients were taking medications to lower blood pressure (68.5%), lipids (43.2%) or glucose levels (23.4%). Since we do not have pre-medication data collected under standardized conditions, we could not calculate the full impact of risk factors or the benefits of clinical care in terms of risk reduction. Third, while the cohorts used to derive the original SCORE equation included participants with diabetes, the equation did not incorporate diabetes as a predictor due to differences in diabetes definition and ascertainment across cohorts [5]. Since the prevalence of diabetes in the EURIKA population was much higher than the prevalence of diabetes in the SCORE populations, we considered that it was important to account for diabetes as an independent risk factor and thus increased the risk of patients with diabetes by a factor derived from a large pooled analysis of diabetes risk [8]. This approach may result in some overestimation of overall risk and of the contribution of diabetes in our study, but is likely a better approximation to the true underlying risk than the original SCORE equation in a population with high diabetes prevalence.

Finally, the SCORE equation is restricted to predict 10-year risk of fatal CVD, and thus underestimate the burden of CVD by excluding non-fatal events and events occurring after 10 years of follow-up. Even with these sources of uncertainty, our analyses indicate that 4 well-established, preventable and controllable risk factors were responsible for almost 60% of estimated CVD risk and that lack of control of these risk factors according to clinical guidelines was responsible for almost 30% of CVD risk, a failure of both clinical medicine and public health.

## Conclusions

In the EURIKA study, lack of control of CVD risk factors was responsible for almost 30% of CVD mortality risk. Systematic monitoring of CVD risk factor levels and of SCORE risk estimates can thus help practitioners understand the implications of risk factor management and control in their patient populations. Patients with high estimated risk, patients with diabetes, elderly patients and current smokers would show substantial absolute reductions in estimated risk by attaining target control levels. These findings are in agreement with current guidelines to direct intensive therapy to patients at high estimated risk and validate the use of SCORE or other risk equations to manage risk reduction [1,2]. The clinical approach to patients with low absolute risk, including young patients with high levels of risk factors, is more complex. Our data indicate that both a clinical and a public health approach are needed to maximize CVD prevention and call for added efforts to develop translational approached that effectively implement prevention strategies in individual patients and in population settings.

## Competing interests

E Guallar has received research grants from AstraZeneca. JP Halcox and J Dallongeville have received speaker fees and consulting fees from AstraZeneca. PG Steg reports receiving research grants from Servier; speaking or consulting fees from Astellas, AstraZeneca, Bayer, Boehringer Ingelheim, Bristol-Myers Squibb, Daiichi-Sankyo, Endotis, Glaxo Smith Kline, Menarini, Medtronic, Merck-Sharpe & Dohme, Otsuka, Pierre Fabre, Hoffmann-La Roche, Sanofi-Aventis, Servier and The Medicines Company, and is a stockholder in Aterovax. EL Massó-González, M Tafalla and FJ Jimenez are employees of AstraZeneca. The rest of authors declare that they have no competing interests.

## Authors' contributions

EG, JRB, FJJ and FRA conceived of the study, and participated in its design and coordination and helped to draft the manuscript. EG carried out the statistical analyses. All authors contributed to and approved the final manuscript.

## Pre-publication history

The pre-publication history for this paper can be accessed here:

http://www.biomedcentral.com/1471-2458/11/704/prepub

## Supplementary Material

Additional file 1 **Tables S1, S2, S3**. Table S1. Absolute excess risk due to traditional cardiovascular risk factors (%), EURIKA Study 2009 - 2010. Table S2. Absolute excess risk due to lack of control of traditional cardiovascular risk factors (%), EURIKA Study 2009 - 2010. Table S3. Cardiovascular risk attributable to traditional cardiovascular risk factors by patient characteristics, EURIKA Study 2009 - 2010. Click here for file
