# Supplementary material for: Excess risk attributable to traditional cardiovascular risk factors in clinical practice settings across Europe - The EURIKA Study
Source: BMC Public Health. 2011 Sep 18;11:704. doi: 10.1186/1471-2458-11-704 (PMC3184074; doi:10.1186/1471-2458-11-704)
Supplement: Additional file 1 — Tables S1, S2, S3. Table S1. Absolute excess risk due to traditional cardiovascular risk factors (%), EURIKA Study 2009 - 2010. Table S2. Absolute excess risk due to lack of control of traditional cardiovascular risk factors (%), EURIKA Study 2009 - 2010. Table S3. Cardiovascular risk attributable to traditional cardiovascular risk factors by patient characteristics, EURIKA Study 2009 - 2010. [file 1471-2458-11-704-S1.DOC]

**Table S1. Absolute excess risk due to traditional cardiovascular risk factors (%), EURIKA Study 2009 – 2010.**

|  |  |  |  |  |  |  |  |  |  |  |  |  |  |  |
| --- | --- | --- | --- | --- | --- | --- | --- | --- | --- | --- | --- | --- | --- | --- |
|  |  |  | **Low risk** | **countries** |  |  |  |  |  | **High risk** | **countries** |  |  |  |
|  |  |  |  |  |  |  |  |  |  |  |  |  |  |  |
|  | **Overall** | **Belgium** | **France** | **Greece** | **Spain** | **Switzerland** |  | **Austria** | **Germany** | **Norway** | **Russia** | **Sweden** | **Turkey** | **UK** |
|  |  |  |  |  |  |  |  |  |  |  |  |  |  |  |
| **Hypertension** | 3.49 | 1.74 | 1.88 | 2.10 | 2.48 | 2.89 |  | 4.33 | 4.92 | 3.96 | 4.57 | 4.91 | 4.29 | 3.67 |
|  | (3.34-3.63) | (1.24-2.24) | (1.34-2.42) | (1.58-2.61) | (1.97-2.98) | (2.40-3.78) |  | (3.81-4.84) | (4.42-5.43) | (3.44-4.49) | (4.08-5.06) | (4.39-5.43) | (3.80-4.77) | (3.17-4.16) |
|  |  |  |  |  |  |  |  |  |  |  |  |  |  |  |
| **Dyslipidemia** | 1.14 | 0.43 | 0.72 | 0.77 | 0.78 | 0.75 |  | 1.67 | 1.76 | 1.48 | 1.74 | 1.55 | 1.30 | 0.73 |
|  | (1.08-1.19) | (0.23-0.62) | (0.51-0.92) | (0.57-0.97) | (0.59-0.98) | (0.56-0.93) |  | (1.47-1.87) | (1.57-1.96) | (1.27-1.68) | (1.55-1.93) | (1.34-1.74) | (1.11-1.48) | (0.54-0.92) |
|  |  |  |  |  |  |  |  |  |  |  |  |  |  |  |
| **Smoking** | 0.92 | 0.52 | 0.38 | 0.99 | 0.44 | 0.83 |  | 1.04 | 1.01 | 1.59 | 1.20 | 1.05 | 1.07 | 0.89 |
|  | (0.85-0.99) | (0.27-0.78) | (0.11-0.66) | (0.73-1.25) | (0.19-0.70) | (0.59-1.07) |  | (0.78-1.30) | (0.76-1.27) | (1.33-1.86) | (0.94-1.46) | (0.79-1.32) | (0.83-1.31) | (0.64-1.14) |
|  |  |  |  |  |  |  |  |  |  |  |  |  |  |  |
| **Diabetes** | 2.25 | 1.32 | 1.09 | 1.73 | 1.68 | 2.06 |  | 2.83 | 3.93 | 2.17 | 2.69 | 2.43 | 3.28 | 1.67 |
|  | (2.12-2.39) | (0.84-1.80) | (0.57-1.60) | (1.24-2.22) | (1.20-2.16) | (1.60-2.52) |  | (2.34-3.32) | (3.45-4.41) | (1.67-2.67) | (2.22-3.17) | (1.94-2.92) | (2.82-3.74) | (1.20-2.14) |
|  |  |  |  |  |  |  |  |  |  |  |  |  |  |  |
| **All risk factors** | 5.66 | 3.06 | 3.09 | 4.02 | 3.99 | 4.65 |  | 7.07 | 8.12 | 6.68 | 7.29 | 7.33 | 7.08 | 5.39 |
|  | (5.47-5.85) | (2.38-3.74) | (2.36-3.82) | (3.32-4.72) | (3.30-4.67) | (4.00-5.30) |  | (6.37-7.77) | (7.44-8.80) | (5.96-7.39) | (6.62-7.97) | (6.63-8.04) | (6.43-7.73) | (4.72-6.06) |
|  |  |  |  |  |  |  |  |  |  |  |  |  |  |  |

Values in Table are % (95% confidence interval).

**Table S2. Absolute excess risk due to lack of control of traditional cardiovascular risk factors (%), EURIKA Study 2009 – 2010.**

|  |  |  |  |  |  |  |  |  |  |  |  |  |  |  |
| --- | --- | --- | --- | --- | --- | --- | --- | --- | --- | --- | --- | --- | --- | --- |
|  |  |  | **Low risk** | **countries** |  |  |  |  |  | **High risk** | **countries** |  |  |  |
|  |  |  |  |  |  |  |  |  |  |  |  |  |  |  |
|  | **Overall** | **Belgium** | **France** | **Greece** | **Spain** | **Switzerland** |  | **Austria** | **Germany** | **Norway** | **Russia** | **Sweden** | **Turkey** | **UK** |
|  |  |  |  |  |  |  |  |  |  |  |  |  |  |  |
| **Hypertension** | 1.42 | 0.45 | 0.55 | 0.69 | 1.01 | 1.24 |  | 1.92 | 2.21 | 1.47 | 1.88 | 2.15 | 1.94 | 1.43 |
|  | (1.31-1.53) | (0.06-0.84) | (0.12-0.97) | (0.28-1.09) | (0.62-1.41) | (0.85-1.62) |  | (1.52-2.33) | (1.82-2.60) | (1.06-1.89) | (1.49-2.26) | (1.74-2.55) | (1.56-2.33) | (1.04-1.82) |
|  |  |  |  |  |  |  |  |  |  |  |  |  |  |  |
| **Dyslipidemia** | 0.92 | 0.33 | 0.56 | 0.62 | 0.61 | 0.61 |  | 1.35 | 1.46 | 1.15 | 1.41 | 1.25 | 1.04 | 0.65 |
|  | (0.87-0.96) | (0.16-0.50) | (0.38-0.74) | (0.45-0.79) | (0.44-0.77) | (0.44-0.77) |  | (1.18-1.52) | (1.30-1.63) | (0.97-1.32) | (1.24-1.58) | (1.08-1.42) | (0.88-1.20) | (0.49-0.81) |
|  |  |  |  |  |  |  |  |  |  |  |  |  |  |  |
| **Smoking** | 0.92 | 0.52 | 0.38 | 0.99 | 0.44 | 0.83 |  | 1.04 | 1.01 | 1.59 | 1.20 | 1.05 | 1.07 | 0.89 |
|  | (0.85-0.99) | (0.27-0.78) | (0.11-0.66) | (0.73-1.25) | (0.19-0.70) | (0.59-1.07) |  | (0.78-1.30) | (0.76-1.27) | (1.33-1.86) | (0.94-1.46) | (0.79-1.32) | (0.83-1.31) | (0.64-1.14) |
|  |  |  |  |  |  |  |  |  |  |  |  |  |  |  |
| **Diabetes** | 0.41 | 0.18 | 0.18 | 0.30 | 0.33 | 0.22 |  | 0.49 | 0.49 | 0.38 | 0.54 | 0.54 | 0.96 | 0.30 |
|  | (0.37-0.46) | (0.04-0.33) | (0.03-0.34) | (0.16-0.45) | (0.18-0.47) | (0.07-0.37) |  | (0.34-0.64) | (0.34-0.63) | (0.23-0.53) | (0.40-0.68) | (0.39-0.68) | (0.81-1.11) | (0.16-0.44) |
|  |  |  |  |  |  |  |  |  |  |  |  |  |  |  |
| **All risk factors** | 3.12 | 1.33 | 1.46 | 2.19 | 2.07 | 2.50 |  | 4.06 | 4.41 | 3.86 | 4.23 | 4.26 | 4.17 | 2.87 |
|  | (2.97-3.27) | (0.80-1.86) | (0.89-2.03) | (1.64-2.73) | (1.53-2.60) | (1,98-3.01) |  | (3.51-4.61) | (3.88-4.94) | (3.30-4.41) | (3.70-4.75) | (3.71-4.81) | (3.66-4.69) | (2.35-3.40) |
|  |  |  |  |  |  |  |  |  |  |  |  |  |  |  |

Values in Table are % (95% confidence interval).

**Table S3. Cardiovascular risk attributable to traditional cardiovascular risk factors by patient characteristics, EURIKA Study 2009 – 2010.**

|  |  |  |  |  |  |  |  |
| --- | --- | --- | --- | --- | --- | --- | --- |
|  |  | **Average 10-y** | **Absolute excess** | **risk, % (95% CI)** |  | **Attributable risk, %** | **(95% CI)** |
|  | **N (%)** | **risk of CVD death, %** | **Presence of** | **Lack of control of** |  | **Presence of** | **Lack of control of** |
|  |  | **(SD)** | **risk factors** | **risk factors** |  | **risk factors** | **risk factors** |
|  |  |  |  |  |  |  |  |
| **Age** |  |  |  |  |  |  |  |
| **<65 y** | 4,396 (59.1) | 4.12 (4.35) | 2.82 (2.57-3.07) | 1.74 (1.55-1.93) |  | 57.2 (56.4-58.0) | 31.4 (30.6-32.2) |
| **≥65 y** | 3,038 (40.9) | 14.17 (12.15) | 9.76 (9.47-10.05) | 5.12 (4.89-5.34) |  | 58.5 (57.5-59.4) | 25.9 (25.0-26.8) |
|  |  |  |  |  |  |  |  |
| **Sex** |  |  |  |  |  |  |  |
| **Men** | 3,584 (48.2) | 9.84 (9.81) | 6.95 (6.69-7.20) | 3.95 (3.75-4.15) |  | 57.8 (56.9-58.6) | 29.7 (28.9-30.6) |
| **Women** | 3,850 (51.8) | 6.73 (9.54) | 4.47 (4.22-4.71) | 2.36 (2.16-2.55) |  | 57.6 (56.8-58.5) | 28.6 (27.8-29.5) |
|  |  |  |  |  |  |  |  |
| **Country** |  |  |  |  |  |  |  |
| **Low-risk** | 3,067 (41.3) | 6.36 (7.60) | 3.78 (3.48-4.09) | 1.92 (1.68-2.16) |  | 55.4 (54.3-56.5) | 26.6 (25.6-27.7) |
| **High-risk** | 4,364 (58.7) | 9.54 (10.89) | 6.99 (6.74-7.25) | 3.98 (3.78-4.18) |  | 59.4 (58.5-60.3) | 31.0 (30.1-31.9) |
|  |  |  |  |  |  |  |  |
| **Individual risk** |  |  |  |  |  |  |  |
| **<5%** | 3,690 (49.6) | 2.37 (1.32) | 1.79 (1.54-2.04) | 0.30 (0.09-0.51) |  | 38.7 (37.9-39.5) | 12.0 (11.2-12.8) |
| **5-10%** | 1,824 (24.5) | 7.15 (1.40) | 4.18 (3.89-4.46) | 2.22 (1.98-2.46) |  | 65.1 (64.2-66.0) | 34.1 (33.1-35.0) |
| **≥10%** | 1,920 (25.8) | 20.51 (12.18) | 14.50 (14.15-14.86) | 9.41 (9.11-9.70) |  | 87.2 (86.1-88.2) | 57.4 (56.3-58.6) |
|  |  |  |  |  |  |  |  |
| **Hypertension** |  |  |  |  |  |  |  |
| **No** | 1,450 (19.5) | 3.82 (4.41) | 3.77 (3.39-4.15) | 2.29 (1.99-2.60) |  | 37.2 (36.1-38.3) | 27.2 (26.0-28.5) |
| **Yes** | 5,984 (80.5) | 9.30 (10.42) | 6.12 (5.91-6.32) | 3.33 (3.16-3.49) |  | 62.6 (62.0-63.3) | 29.6 (28.9-30.3) |
|  |  |  |  |  |  |  |  |
| **Dyslipidemia** |  |  |  |  |  |  |  |
| **No** | 788 (10.6) | 7.37 (8.20) | 3.53 (3.03-4.03) | 1.52 (1.12-1.92) |  | 47.3 (45.7-48.9) | 19.4 (17.8-21.0) |
| **Yes** | 6,646 (89.4) | 8.33 (9.96) | 5.92 (5.72-6.11) | 3.32 (3.16-3.47) |  | 58.9 (58.2-59.6) | 30.3 (29.7-31.0) |
|  |  |  |  |  |  |  |  |
| **Current smoking** |  |  |  |  |  |  |  |
| **No** | 5,846 (78.6) | 8.05 (9.47) | 4.73 (4.52-4.94) | 2.16 (1.99-2.32) |  | 53.3 (52.6-54.0) | 21.1 (20.5-21.7) |
| **Yes** | 1,588 (21.4) | 8.90 (10.90) | 9.09 (8.73-9.45) | 6.69 (6.41-6.97) |  | 73.8 (72.7-75.0) | 58.9 (58.0-59.9) |
|  |  |  |  |  |  |  |  |
| **Diabetes** |  |  |  |  |  |  |  |
| **No** | 5,234 (70.4) | 5.88 (6.37) | 3.63 (3.43-3.82) | 2.03 (1.87-2.19) |  | 49.7 (49.1-50.3) | 26.1 (25.3-26.8) |
| **Yes** | 2,200 (29.6) | 13.82 (13.54) | 10.52 (10.24-10.81) | 5.73 (5.50-5.97) |  | 76.9 (76.1-77.7) | 36.6 (35.6-37.6) |
|  |  |  |  |  |  |  |  |
